# Predictive Threshold Value of the Breathing Reserve for the Decline in Cardiorespiratory Fitness Among the Healthy Middle-Aged Population

**DOI:** 10.3390/jcdd12030085

**Published:** 2025-02-24

**Authors:** Tao Shen, Yang Wang, Jinglin Li, Shunlin Xu, Peng Wang, Wei Zhao

**Affiliations:** 1 NHC Key Laboratory of Cardiovascular Molecular Biology and Regulatory Peptides, Key Laboratory of Molecular Cardiovascular Science, Ministry of Education, Department of Cardiology, Peking University Third Hospital, Beijing 100191, China; fengguxuan@163.com (T.S.); lijinglin@foxmail.com (J.L.); shunlinxu@163.com (S.X.); 2 Physical Examination Center, Peking University Third Hospital, Beijing 100191, China; bysywyy@163.com

**Keywords:** cardiorespiratory fitness, breathing reserve, work of breathing

## Abstract

Objective: To investigate the cut-off value of the breathing reserve for predicting a decline in cardiorespiratory fitness (CRF) among healthy middle-aged Chinese individuals. Methods: Healthy middle-aged individuals who underwent cardiopulmonary exercise testing (CPET) at the Peking University Third Hospital from May to October 2021 were selected. The study included 321 participants, with an average age of 48.8 ± 5.7 years. They were divided into two groups based on the peak oxygen uptake (VO_2_peak): the adequate CRF group and the CRF decline group. Multivariate logistic regression analysis was used to explore the factors influencing CRF. Results: In the male CRF decline group, heart rate, alanine aminotransferase, end-tidal partial pressure of carbon dioxide (PETCO_2_), and breathing reserve (BR%) were significantly higher, while the oxygen uptake at the anaerobic threshold (VO_2_@AT) was lower. An elevated BR% was independently associated with CRF decline (OR = 1.111, 95% CI: 1.068–1.156). The female CRF decline group had significantly higher FEV1/FVC and BR% and significantly lower age, fasting glucose, hemoglobin, and VO_2_@AT compared to the adequate CRF group. Elevated BR% was independently associated with CRF decline (OR = 1.086, 95% CI: 1.038–1.137). The receiver operating characteristic (ROC) curve for the males showed an area under the curve (AUC) of 0.769 (95% CI: 0.703–0.827) with an appropriate BR% cut-off value of 49.9%, sensitivity of 59.9%, and specificity of 77.8%. For the females, the ROC curve displayed an AUC of 0.694 (95% CI: 0.607–0.773) with an appropriate BR% cut-off value of 57.0%, sensitivity of 58.7%, and specificity of 86.0%. Conclusions: The breathing reserve was independently associated with CRF. The appropriate cut-off values for BR% to predict CRF decline were 49.9% for the males and 57.0% for the females.

## 1. Introduction

Cardiorespiratory fitness (CRF) represents the ability of the circulatory, respiratory, and muscular systems to absorb, transport, and utilize oxygen during physical activity [1]. It is one of the most critical health indicators in both healthy populations and individuals at an increased risk of cardiovascular diseases. The determinants of CRF include central factors, such as pulmonary gas exchange, respiratory function, maximum cardiac output, and blood oxygen-carrying capacity, as well as peripheral factors, such as muscle oxygen uptake and mitochondrial transport of oxygen [2].

Breathing reserve (BR%) is a measure used to evaluate residual lung function at peak exercise. It is assessed by comparing the peak minute ventilation (VE) during CRF testing with the maximum voluntary ventilation (MVV) at rest. A decrease in BR% can indicate high ventilatory demand or reduced ventilatory capacity, with the BR < 30% during exercise generally considered abnormal. Conditions such as chronic obstructive pulmonary disease (COPD) can reduce the gap between the peak VE and the MVV, leading to a lower BR% and manifesting as exercise-induced ventilatory limitation [3,4]. Conversely, an increased BR% may be observed in individuals whose exercise capacity is restricted due to cardiovascular diseases or other health conditions [5].

This study aims to explore the relationship between BR% and the peak oxygen uptake (VO_2_peak) in populations without chronic lung diseases, to determine the optimal range of BR%, and to establish the appropriate cut-off values for predicting CRF decline in middle-aged Chinese adults.

## 2. Materials and Methods

### 2.1. Study Design

For this study, healthy individuals undergoing cardiopulmonary exercise testing (CPET) at the Health Management (Physical Examination) Center of Peking University Third Hospital from May 2021 to October 2021 were selected.

### 2.2. Inclusion and Exclusion Criteria of the Participants

**Figure 1:**
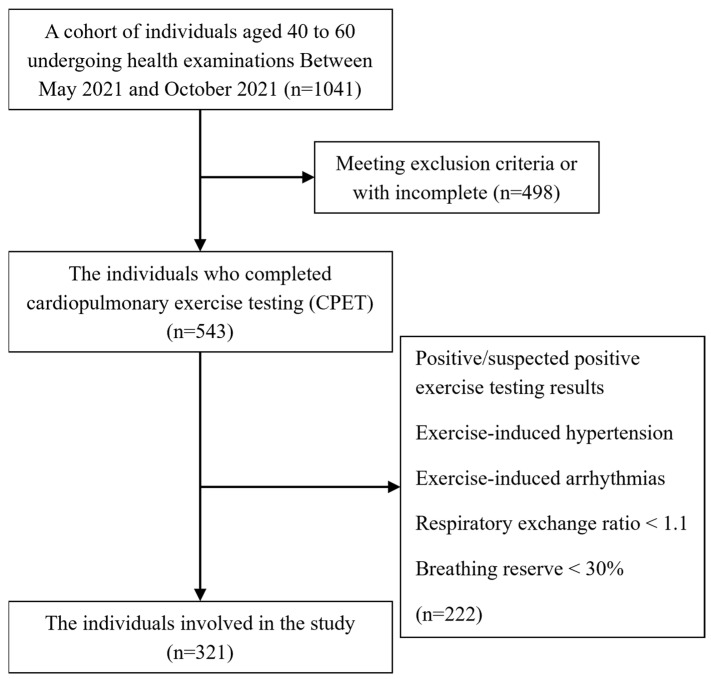
Flowchart of the study participants.

As shown in Figure 1, the inclusion criteria for this study were age between 40 and 60 years, completion of CPET with a respiratory exchange ratio (RER) ≥ 1.1, BR% ≥ 30%, and complete biochemical data. The exclusion criteria included (1) the presence of infectious diseases; (2) presence of coronary heart disease, heart failure, pulmonary hypertension, valvular disease, cardiomyopathy, severe ventricular arrhythmia, myocarditis, or pericarditis; (3) presence of severe respiratory diseases, such as chronic obstructive pulmonary disease or severe asthma; (4) uncontrolled hyperthyroidism or hypothyroidism; (5) severe anemia; (6) severe electrolyte disturbances; (7) severe hypertension; (8) uncontrolled diabetes; (9) abnormal liver function, renal function, or myocardial enzymes; (10) abnormal findings in electrocardiograms, echocardiography, or carotid ultrasound; (11) absolute contraindications to exercise testing; (12) abnormal cardiovascular responses during exercise.

### 2.3. General Data Collection and Physical Examination

Demographic characteristics and medical history, including hypertension, diabetes, and dyslipidemia, were collected through questionnaires. Physical examination included measurements of the BMI, systolic blood pressure (SBP), diastolic blood pressure (DBP), and heart rate (HR). The BMI was calculated using the values measured with a height–weight scale (HNH-318, Omron Corporation, Shenzhen, China). Grip strength was measured with a grip strength monitor (CSTF-WL, Tongfang Health Technology Co., Ltd., Beijing, China).

Venous blood samples were collected in vacuum blood collection tubes and uniformly sent to the hospital laboratory for analysis. Blood biochemical indicators, including fasting glucose (Glu), total cholesterol (TC), low-density lipoprotein cholesterol (LDL-C), triglycerides (TG), high-density lipoprotein cholesterol (HDL-C), hemoglobin (Hb), and high-sensitivity C-reactive protein (CRP), were measured using an automatic biochemical analyzer (Beckman Coulter Inc., Brea, CA, USA). Liver function indicators included alanine aminotransferase (ALT) and aspartate aminotransferase (AST). Renal function indicators included estimated glomerular filtration rate (eGFR), creatinine, uric acid, and homocysteine (Hcy).

### 2.4. Cardiopulmonary Exercise Testing

Ventilation and expired gases were sequentially measured using COSMED equipment (Quark PFT Ergo, Rome, Italy), and all measurement data were analyzed as 20-s averages. Before testing, the purpose of the test and the procedures were explained to the subjects, and they were familiarized with the Borg Rating of Perceived Exertion scale.

Pulmonary function was measured in a sitting position, with forced vital capacity (FVC), forced expiratory volume in one second (FEV1), and maximum voluntary ventilation (MVV) measured using a spirometer. FEV1 was measured three times, and the maximum value was selected. All the standards were in accordance with the recommendations of the American Thoracic Society/European Respiratory Society [6].

Before measurement, the subjects were instructed to breathe normally three times and then inhale and exhale as quickly as possible while making maximum effort each time to fully expand the lungs. The cycle ergometer was adjusted to the most suitable position according to age, gender, height, weight, and predicted functional status. Dynamic electrocardiogram, blood pressure, and pulse oxygen saturation were monitored during rest and exercise. The mask was checked for air leaks, and the instrument was worn properly.

The exercise protocol included 3 min of sitting rest on the cycle ergometer, 3 min of warm-up exercise at a rate of 60 revolutions per minute without power load, followed by a ramp protocol to complete the incremental exercise test within 8 to 12 min. Termination criteria included reaching the target heart rate; experiencing angina pectoris; experiencing neurological symptoms such as ataxia or dizziness; showing signs of poor perfusion such as cyanosis or pallor; a significant drop in blood pressure ≥ 10 mmHg or a significant increase ≥ 210 mmHg; abnormal electrocardiogram findings such as ST segment elevation or depression ≥ 0.1 mV, or persistent tachycardia; and an inability to continue by the subject.

Post-test, the subjects were monitored under rest conditions with ECG for at least six minutes or until recovery to the pre-exercise state. Borg ratings for dyspnea and general fatigue were recorded [7]. Minute ventilation (VE), oxygen uptake (VO_2_), carbon dioxide output (VCO_2_), and RER were recorded. The predicted VO_2_peak was calculated according to the Wasserman formula. The subjects were divided into the target group (VO_2_peak % ≥ 85%) and the decline group (VO_2_peak % < 85%) based on their VO_2_peak as a percentage of the predicted value. Breathing reserve (BR%) was calculated as [(MVV − VEpeak) × 100/MVV].

### 2.5. Statistical Methods

The continuous variables were tested for normal distribution using the Kolmogorov–Smirnov test. Skewed continuous variables are presented as the medians (interquartile ranges [IQRs]). The categorical variables are presented as the absolute numbers or percentages. Comparisons of the continuous variables were conducted using the t-test or Mann–Whitney U test, while comparisons of the categorical variables were performed using the chi-squared test or Fisher’s exact test.

Univariate (unadjusted) and multivariate (adjusted) logistic regression analyses were utilized to calculate the clinical variables associated with a decline in CRF, yielding odds ratios (ORs) and 95% confidence intervals (CIs). Significant predictors from the univariate analysis (*p* < 0.05) were included in the multivariate analysis.

The suitable cut-off value for BR% corresponding to the study results was determined through receiver operating characteristic (ROC) curve analysis, calculating the area under the curve (AUC). The optimal cut-off value was identified using the Youden’s index, and the sensitivity, specificity, negative predictive value (NPV), positive predictive value (PPV), negative likelihood ratio (–LR), and positive likelihood ratio (+LR) were calculated. Two-sided *p*-value < 0.05 was considered statistically significant. All statistical analyses were conducted using SPSS software (version 25.0; IBM, Chicago, IL, USA).

## 3. Results

### 3.1. Characteristics of the Study Subjects

A retrospective analysis ultimately included data from 321 healthy individuals undergoing physical examinations, comprising 193 males (60.1%) and 128 females (39.9%), with an average age of 48.8 ± 5.7 years. The clinical and demographic characteristics of the study participants are presented in Table 1.

### 3.2. Relationship Between the Physical Examination, Biochemical Indicators, Respiratory Function, and Decline in CRF

Univariate logistic regression analysis was used to explore the predictors of a decline in CRF (Table 2).

In the males, the significant predictors for a decline in CRF included HR (OR = 1.041, 95% CI: 1.01–1.072), ALT (OR = 1.029, 95% CI: 1.001–1.057), BR% (OR = 1.111, 95% CI: 1.068–1.155), VO_2_@AT (OR = 0.866, 95% CI: 0.776–0.966), and PETCO_2_@VO_2_peak (OR = 1.198, 95% CI: 1.083–1.326).

In the females, significant predictors for a decline in CRF included age (OR = 0.917, 95% CI: 0.844–0.996), Glu (OR = 0.399, 95% CI: 0.161–0.988), Hb (OR = 0.955, 95% CI: 0.917–0.995), FEV1/FVC (OR = 1.722, 95% CI: 1.027–2.888), BR% (OR = 1.077, 95% CI: 1.032–1.123), and VO_2_@AT (OR = 0.824, 95% CI: 0.689–0.985).

Significant predictors from the univariate analysis were included in the multivariate logistic regression analysis. After adjustment, increased BR% was found to be independently and significantly associated with a decline in CRF in both the males (OR = 1.111, 95% CI: 1.068–1.156) and the females (OR = 1.086, 95% CI: 1.038–1.137).

The ROC analysis results revealed that for the males, the AUC for BR% predicting a decline in CRF was 0.769 (95% CI: 0.703–0.827), with a cut-off value of 49.8% based on Youden’s index, sensitivity of 59.9%, specificity of 77.8%, NPV of 58.8%, PPV of 88.8%, +LR of 3.08, and −LR of 0.41.

**Figure 2:**
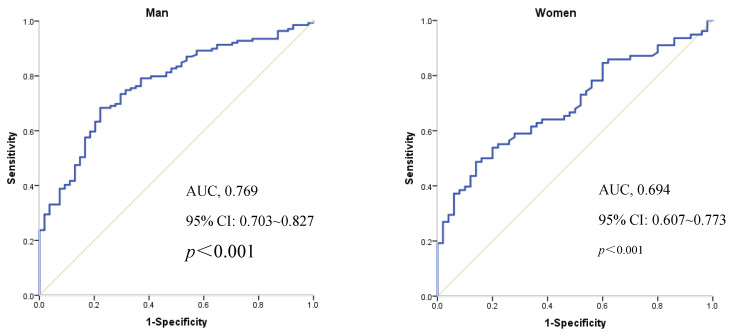
Receiver operating characteristic (ROC) curves for the BR% predicting a decline in CRF by gender.

For the females, the AUC for BR% predicting a decline in CRF was 0.694 (95% CI: 0.607–0.773), with a cut-off value of 57.0% based on Youden’s index, sensitivity of 58.7%, specificity of 86.0%, NPV of 61.8%, PPV of 84.4%, +LR of 3.48, and −LR of 0.60 (Figure 2).

## 4. Discussion

This study analyzed the appropriate cut-off values of BR% for predicting a decline in CRF in healthy individuals of different genders. After adjusting for age, BMI, blood pressure, and lipid profile, the results showed that the BR% cut-off value associated with a decline in CRF was 49.9% for the males (AUC = 0.769, 95% CI: 0.703–0.827) and 57.0% for the females (AUC = 0.694, 95% CI: 0.607–0.773).

Although BR% is an important outcome of CPET, it has not been adequately emphasized for a long time. Aging can lead to structural and functional changes in the respiratory system, such as loss of elastic recoil, increased chest wall stiffness, decreased chest wall compliance, weakened respiratory muscle strength, reduced alveolar surface area, and decreased pulmonary vascular perfusion. These factors can affect ventilation and pulmonary gas exchange, leading to a decline in CRF [8]. In individuals with inadequate exercise, the reduced elastic capacity of ventilation and lung function utilization can result in increased BR%.

### 4.1. Potential Role of BR% in a Decline in CRF

BR% is influenced by several factors, including metabolic demand, body weight, dead space ventilation, neural regulation, and behavioral factors affecting ventilation demand, as well as mechanical factors such as airflow limitation, work volume of the lung, inspiratory muscle function, aging, and disease [9].

Historically, BR% has been considered an indicator of ventilatory limitation, useful for the differential diagnosis of dyspnea related to lung conditions. For patients with COPD, the peak exercise VE close to the MVV can result in a lower BR%. Thus, BR% is crucial for determining whether dyspnea during exertion is related to ventilation [4,10].

Studies have shown a negative correlation between BR% and VO_2_peak, with an increased BR% associated with a decline in CRF, indicating reduced exercise capacity [11]. Healthy individuals may approach the MVV during exercise [12], and a decreased BR% indicates an appropriate ventilation response to an increased workload [3].

### 4.2. Gender-Specific Influencing Factors of BR%

Ventilatory limitations during exercise induced by smaller lung volumes and maximum expiratory flow rates are more common in females, whereas males adopt a more efficient breathing pattern. Females exhibit distinct respiratory system characteristics, including increased mechanical ventilatory limitations with age and training, leading to specific ventilatory adaptations during exercise [13]. Females tend to have relatively smaller lung volumes and airway diameters, resulting in higher airflow resistance compared to males [14,15,16], leading to a higher work of breathing (WOB) for a given VE [13,15,17]. Consequently, females maintain faster and shallower breathing patterns during exercise to sustain adequate alveolar ventilation (VA) [18], which increases dead space ventilation and decreases ventilatory efficiency [19].

Studies have suggested that females’ unique chest structure may reduce reliance on the diaphragm during exercise, mitigating diaphragm fatigue and preserving exercise capacity [20]. The increase in the WOB and mechanical ventilatory limitations in females results in a higher oxygen cost of breathing, necessitating more mechanical work to move gases in and out of the lungs [17].

### 4.3. Reasons for Gender-Specific BR% Cut-Off Values

During high-intensity exercise, respiratory muscles and exercise muscles compete for the blood flow, with the work done by the respiratory muscles reducing the blood flow to the exercise muscles [21]. Female breathing patterns during exercise involve activation of the different muscles, resulting in a different blood flow redistribution compared to males [22].

The mechanism behind blood flow redistribution stems from the sympathetic reflex of the respiratory muscles, and high-intensity exercise leads to the redistribution of the skeletal muscle blood flow [22,23].

In males, respiratory muscle work leads to an increase in muscle sympathetic nerve activity, reflexive increases in blood pressure, and a decrease in limb blood flow [24,25]. However, in females, the respiratory metabolic response is weakened due to a reduction in the sympathetic motor outflow, thereby maintaining the lower limb blood flow and vascular resistance [26,27].

Additionally, estrogen’s influence on the central and peripheral sympathetic nervous systems helps maintain muscle oxygenation and delay fatigue, presenting hemodynamic benefits during exercise [28,29].

### 4.4. Respiratory Training

Weakened respiratory muscle strength affects CRF and exercise performance in healthy individuals [30]. Respiratory training can extend the fatigue time, improve exercise performance, and maintain balance in the arterial blood gases and the acid–base status.

Endurance exercise training, especially combined with specific respiratory muscle training, enhances the respiratory muscle strength and endurance, improving the gas exchange and preventing or alleviating respiratory muscle dysfunction, thus benefiting exercise endurance.

Respiratory training includes diaphragmatic breathing, resistance breathing, inspiratory muscle strength training, and inspiratory muscle endurance training. Inspiratory muscle training through repeated respiratory exercises at certain load intensities improves the strength of the diaphragm and other respiratory muscles, enhancing muscle endurance, reducing exertional dyspnea in health and disease, and improving cardiopulmonary function [31].

Respiratory training improves chest and abdominal movement patterns, with notable benefits for healthy individuals experiencing CRF decline compared to athletes [32,33]. Additionally, respiratory training can reduce blood pressure, heart rate, and respiratory rate [34]. Overall, respiratory training increases the inspiratory muscle strength and endurance, enhances the exercise capacity, alleviates dyspnea symptoms, and improves the cardiopulmonary function.

### 4.5. Limitations

This study had some limitations. Firstly, it was a non-randomized observational study involving a relatively healthy middle-aged population, without considering lifestyle factors such as smoking and exercise that might affect CRF.

Secondly, the MVV measurement adopted a high breathing frequency and small tidal volume rather than the ability to maintain rapid and deep breathing during exercise [35]. Using the MVV as a measurement method for the ventilatory capacity during exercise had its limitations as mechanical differences between resting and breathing and exercise-induced hyperventilation can affect BR% measurement.

Thirdly, the MVV depended on the individual ability to maintain rapid and deep breathing; an insufficient effort may lower the MVV and, subsequently, the calculated BR%. Fourthly, the MVV was measured before exercise testing, which omits other factors that might limit lung mechanical function before reaching the expected BR%, including respiratory muscle fatigue and dynamic hyperinflation due to the expiratory flow limitation. Additionally, some indicators such as oxygen saturation, VE/VO_2_, and PETO_2_, which were not included in this study, may affect the results. Finally, the study included only Chinese subjects and might not be generalized to other races.

The innovative aspect of this study lies in determining the appropriate cut-off values for BR% in predicting CRF decline in both genders; females are more prone to the expiratory flow limitation compared to males. CRF results can provide individualized endurance, resistance, and respiratory training methods, encouraging the maintenance of a healthy lifestyle and guidance for exercise training to improve the quality of life.

## 5. Conclusions

The breathing reserve was independently associated with CRF. The appropriate cut-off values for BR% to predict CRF decline were 49.9% for the males and 57.0% for the females.

## Figures and Tables

**Table 1:** Clinical characteristics of the participants.

		Male (*n* = 193)	Female (*n* = 128)	Total
Demographic information	Age (years)	49.0 (43.0~55.8)	49.0 (44.0~52.0)	48.8 ± 5.7
	BMI (kg/m^2^)	25.2 ± 2.8	22.8 ± 2.4	24.3 ± 2.9
	Grip strength (kg)	38.6 (33.8, 43.3)	24.7 (21.8, 28.0)	33.2 ± 9.4
	SBP (mmHg)	122.5 ± 14.0	115.7 ± 14.9	119.8 ± 14.7
	DBP (mmHg)	79.1 ± 9.3	73.0 ± 11.0	76.7 ± 10.4
	HR (Bpm)	82.8 ± 11.6	80.7 ± 10.5	81.9 ± 11.2
Comorbidity	Hypertension [N (%)]	34 (17.9)	4 (3.2)	38 (12.1)
	Diabetes [N (%)]	6 (3.2)	1 (0.8)	7 (2.2)
	Hyperlipidemia [N (%)]	8 (4.2)	4 (3.2)	12 (3.8)
Biochemical indexes	Glu (mmol/L)	5.2 (4.9, 5.6)	5.0 (4.8, 5.2)	5.2 ± 0.6
	TC (mmol/L)	5.0 ± 0.9	5.1 ± 0.9	5.0 ± 0.9
	LDL-C (mmol/L)	3.0 ± 0.7	3.0 ± 0.7	3.0 ± 0.7
	TG (mmol/L)	1.6 ± 0.9	1.1 ± 0.5	1.4 ± 0.8
	HDL-C (mmol/L)	1.2 ± 0.2	1.4 ± 0.3	1.3 ± 0.3
	Hb (g/L)	156.5 ± 9.7	133.1 ± 9.6	147.2 ± 15.0
	hs-CRP (mg/L)	1.3 ± 2.1	1.2 ± 2.8	1.2 ± 2.4
Liver function	ALT (U/L)	23.0 (17.0, 32.0)	14.0 (11.0, 20.0)	23.6 ± 17.5
	AST (U/L)	24.0 ± 8.0	20.5 ± 6.2	22.6 ± 7.6
Renal function	eGFR [mL/min/1.73 m^2^]	88.2 ± 9.8	89.9 ± 11.3	88.9 ± 10.4
	Creatinine (μmol/L)	89.0 ± 8.9	69.7 ± 7.9	81.3 ± 12.8
	Uric acid (μmol/L)	376.5 (322.0, 432.8)	270.0 (228.3, 313.0)	340.0 ± 91.3
	Hcy (μmol/L)	13.4 (11.6, 16.3)	9.9 (8.6, 11.4)	13.5 ± 6.8
Respiratory function	BR%	51.2 ± 10.8	51.7 ± 9.4	51.4 ± 10.1
	FEV1%pred	94.2 ± 11.7	96.3 ± 10.3	95.0 ± 11.2
	FEV1/FVC (%)	79.6 (78.8, 80.4)	81.2 (80.7, 82.0)	80.3 ± 1.2
	Borg score	16.9 ± 1.0	16.7 ± 1.0	16.8 ± 1.0
	RER	1.2 ± 0.1	1.2 ± 0.1	1.2 ± 0.01
Cardiopulmonary parameters	VO_2_peak (mL/kg/min)	24.8 (21.9, 28.3)	20.4 (17.6, 23.3)	23.3 ± 5.1
	VO_2_peak (L/min)	1.8 (1.6, 2.1)	1.2 (1.0, 1.4)	1.6 ± 0.4
	VO_2_%pred (%)	77.3 ± 13.3	80.4 ± 14.8	78.5 ± 14.0
	VO_2_@AT (mL/min/kg)	15.6 ± 3.7	13.4 ± 3.0	14.7 ± 3.6
	VE/VCO_2_@AT	27.1 ± 3.1	27.8 ± 2.8	27.4 ± 3.0
	Heart rate reserve	11.0 ± 9.1	12.2 ± 8.5	11.8 ± 8.8
	PETCO_2_@VO_2_peak (mmHg)	41.5 ± 5.0	39.7 ± 4.1	40.8 ± 4.7

Abbreviation: BMI, body mass index; HR, heart rate; Glu, fasting glucose; TC, total cholesterol; TG, triglycerides; LDL-C, low-density lipoprotein cholesterol; HDL-C, high-density lipoprotein cholesterol; Hb, hemoglobin; hs-CRP, high-sensitivity C-reactive protein; ALT, alanine transaminase; AST, aspartate transaminase; eGFR, estimated glomerular filtration rate; Hcy, homocysteine; BR%, breathing reserve; FEV1, forced expired volume in 1 s; FVC, forced vital capacity; RER, respiratory exchange ratio; VO_2_peak, peak oxygen uptake; VO_2_@AT, oxygen uptake at the anaerobic threshold; VE/VCO_2_@AT, ventilation per carbon dioxide output at the anaerobic threshold; PETCO_2_, end-tidal partial pressure of carbon dioxide.

**Table 2:** Univariate and multivariate logistic regression analysis of the predictors for the decline in CRF.

		Male (*n* = 193)			Female (*n* = 128)		
		OR	95% CI	*p*-Value	OR	95% CI	*p*-Value
Demographic information	Age (years)	1.005	0.956–1.056	0.855	0.917	0.844–0.996	0.040
	BMI (Kg/m^2^)	0.978	0.875–1.092	0.689	0.958	0.826–1.110	0.569
	Grip strength (Kg)	0.969	0.928–1.011	0.148	0.931	0.860–1.008	0.077
	SBP (mmHg)	0.987	0.966–1.01	0.263	0.992	0.969–1.016	0.510
	DBP (mmHg)	1.002	0.968–1.036	0.923	0.976	0.945–1.009	0.153
	HR (Bpm)	1.041	1.01–1.072	0.008	0.999	0.965–1.033	0.941
Biochemical indices	Glu (mmol/L)	1.084	0.669–1.755	0.743	0.399	0.161–0.988	0.047
	TC (mmol/L)	1.317	0.925–1.876	0.126	1.027	0.696–1.515	0.894
	LDL-C (mmol/L)	1.294	0.839–1.997	0.244	0.916	0.564–1.488	0.723
	TG (mmol/L)	1.245	0.844–1.835	0.269	0.671	0.336–1.340	0.258
	HDL-C (mmol/L)	1.433	0.46–4.465	0.535	2.223	0.708–6.978	0.171
	Hb (g/L)	1.004	0.972–1.038	0.795	0.955	0.917–0.995	0.028
	hs-CRP (mg/L)	1.068	0.893–1.277	0.47	1.022	0.889–1.175	0.758
Liver function	ALT (U/L)	1.029	1.001–1.057	0.045	1.013	0.976–1.052	0.488
	AST (U/L)	1.038	0.987–1.091	0.148	1.012	0.954–1.073	0.699
Renal function	eGFR [mL/min/1.73 m^2^]	0.983	0.951–1.016	0.302	1.027	0.994–1.060	0.110
	Creatinine (μmol/L)	1.019	0.983–1.056	0.296	0.970	0.927–1.016	0.195
	Uric acid (μmol/L)	0.998	0.994–1.002	0.343	0.997	0.991–1.003	0.336
	Hcy (μmol/L)	0.964	0.926–1.004	0.075	0.975	0.892–1.066	0.585
Respiratory function	FEV1%pred	0.975	0.949–1.002	0.071	0.970	0.936–1.006	0.097
	FEV1/FVC (%)	0.966	0.678–1.378	0.85	1.722	1.027–2.888	0.039
	BR%	1.111	1.068–1.155	<0.001	1.077	1.032–1.123	0.001
	Borg score	0.776	0.549–1.096	0.150	0.645	0.408–1.022	0.062
	RER	0.612	0.415–1.854	0.827	0.634	0.463–1.893	0.483
Cardiopulmonary parameters	VO_2_@AT (mL/min/Kg)	0.866	0.776–0.966	0.010	0.824	0.689–0.985	0.034
	VE/VCO_2_@AT	1.113	0.941–1.317	0.210	0.960	0.777–1.186	0.707
	Heart rate reserve	1.022	0.983–1.064	0.276	1.047	0.992–1.106	0.098
	PETCO_2_@VO_2_peak (mmHg)	1.198	1.083–1.326	<0.001	1.087	0.947–1.247	0.237
Multivariable	BR%	1.111	1.068–1.156	<0.001	1.086	1.038–1.137	<0.001
analysis	Age, years	-	-	-	0.895	0.817–0.981	0.017

Abbreviation: BMI, body mass index; HR, heart rate; Glu, fasting glucose; TC, total cholesterol; TG, triglycerides; LDL-C, low-density lipoprotein cholesterol; HDL-C, high-density lipoprotein cholesterol; Hb, hemoglobin; hs-CRP, high-sensitivity C-reactive protein; ALT, alanine transaminase; AST, aspartate transaminase; eGFR, estimated glomerular filtration rate; Hcy, homocysteine; BR%, breathing reserve; FEV1, forced expired volume in 1 s; FVC, forced vital capacity; RER, respiratory exchange ratio; VO_2_peak, peak oxygen uptake; VO_2_@AT, oxygen uptake at the anaerobic threshold; VE/VCO_2_@AT, ventilation per carbon dioxide output at the anaerobic threshold; PETCO_2_, end-tidal partial pressure of carbon dioxide.

## Data Availability

The data that support the findings of this study are available upon reasonable request from the corresponding author.
